# Low b-value diffusion weighted imaging is promising in the diagnosis of brain death and hypoxic-ischemic injury secondary to cardiopulmonary arrest

**DOI:** 10.1186/s13054-018-2087-9

**Published:** 2018-06-20

**Authors:** Miriam E. Peckham, Jeffrey S. Anderson, Ulrich A. Rassner, Lubdha M. Shah, Peter J. Hinckley, Adam de Havenon, Seong-Eun Kim, J. Scott McNally

**Affiliations:** 1 Department of Radiology and Imaging Sciences, University of Utah, Salt Lake City, UT USA; 2 Department of Neurology, University of Utah, Salt Lake City, UT USA; 3 Utah Center for Advanced Imaging Research, Department of Radiology, University of Utah, Salt Lake City, UT USA; 4 Department of Radiology and Imaging Sciences, University of Utah Health Sciences Center, 30 North, 1900 East #1A071, Salt Lake City, UT 84132-2140 USA

**Keywords:** Brain death, Hypoxic ischemic encephalopathy, Cardiorespiratory arrest, Diffusion imaging

## Abstract

**Background:**

Cardiorespiratory arrest can result in a spectrum of hypoxic ischemic brain injury leading to global hypoperfusion and brain death (BD). Because up to 40% of patients with BD are viable organ donors, avoiding delayed diagnosis of this condition is critical. High b-value diffusion weighted imaging (DWI) measures primarily molecular self-diffusion; however, low b-values are sensitive to perfusion. We investigated the feasibility of low b-value DWI in discriminating the global hypoperfusion of BD and hypoxic ischemic encephalopathy (HIE).

**Methods:**

We retrospectively reviewed cardiorespiratory arrest subjects with a diagnosis of HIE or BD. Inclusion criteria included brain DWI acquired at both low (50 s/mm^2^) and high (1000–2000 s/mm^2^) b-values. Automated segmentation was used to determine mean b50 apparent diffusion coefficient (ADC) values in gray and white matter regions. Normal subjects with DWI at both values were used as age- and sex-matched controls.

**Results:**

We evaluated 64 patients (45 with cardiorespiratory arrest and 19 normal). Cardiorespiratory arrest patients with BD had markedly lower mean b50 ADC in gray matter regions compared with HIE (0.70 ± 0.18 vs. 1.95 ± 0.25 × 10^−3^ mm^2^/s, *p* < 0.001) and normal subjects (vs. 1.79 ± 0.12 × 10^−3^ mm^2^/s, *p* < 0.001). HIE had higher mean b50 ADC compared with normal (1.95 ± 0.25 vs. 1.79 ± 0.12 × 10^−3^ mm^2^/s, *p* = 0.016). There was wide separation of gray matter ADC values in BD subjects compared with age matched normal and HIE subjects. White matter values were also markedly decreased in the BD population, although they were less predictive than gray matter.

**Conclusion:**

Low b-value DWI is promising for the discrimination of HIE with maintained perfusion and brain death in cardiorespiratory arrest.

## Background

Evaluation of brain perfusion is critically important in patients after cardiorespiratory arrest. These patients suffer an initial hypoxic ischemic injury which can lead to cytotoxic edema as well as global hypoperfusion and brain death (BD). In these patients, ancillary tests can evaluate perfusion status when the clinical examination is limited [1]. Over 40% of subjects with BD are viable organ donors; however, only 10% of subjects with cardiac arrest go on to BD. Because delayed diagnosis of BD has been found to contribute to the overall shortage of viable organ transplants, some have argued for earlier use of ancillary testing in subjects where examination in this critical diagnosis is obscured by central nervous system (CNS) depressant medication [2, 3].

Introduction of targeted temperature management has significantly improved the clinical course in comatose cardiorespiratory arrest patients, with some patients being discharged with minimal brain damage. However, neurologic assessment during this time has remained a critical barrier since sedation and muscle paralysis from hypothermia obscure the clinical examination in the first 24–48 h, a crucial time for these patients [4, 5]. It is suspected that sedation-obscured clinical examination may contribute to withdrawal of life-sustaining therapy in up to 20% of patients during this time who may have otherwise had complete neurologic recovery [4, 5]. Conversely, CNS depressant medications have also been linked to a delay in prognosis of patients with BD, which has negative implications for organ transplantation viability and has led to increased mortality in cardiac transplant recipients [2]. These two extremes demonstrate the clinical dilemma in these unresponsive patients under hypothermic therapy or CNS depressants: the discrimination between subjects sustaining global injury with maintained cerebral blood flow, and subjects with cessation of blood flow and irreversible loss of function defined as BD [6].

Single photon emission computed tomography (SPECT) nuclear scintigraphy is an accepted ancillary test for BD [7–12]. Transcranial Doppler has also been used as a prognostic tool, showing poor outcomes for patients with hypoperfusion, and good prediction of survivability in patients with normal perfusion [13, 14]. Computed tomography angiography (CTA) has been found to be comparable to ancillary tests, but lacks sensitivity [1]. In magnetic resonance imaging (MRI), the presence of bilateral transcerebral and cortical vein signs on susceptibility-weighted imaging (SWI) are helpful indicators in patients with both BD and hypoxic ischemic encephalopathy (HIE), although they are not specific [7, 15]. MRI in BD also consistently shows loss of vascular flow-voids on T2 spin echo and flow-related enhancement on magnetic resonance angiography (MRA), and diffuse cerebral edema with sulcal effacement [7]. Arterial spin labeling (ASL) has been validated as an effective noncontrast method for demonstrating perfusion deficits by demonstrating global hypoperfusion in BD with diffusely impaired cerebral blood flow on visual and quantitative analysis [16, 17]. Diffusion weighted imaging (DWI) shows promise, and apparent diffusion coefficient (ADC) values obtained at high gradient strengths (b750–2000) have been shown to be helpful prognostic indicators in patients with HIE, with decreased ADC values predicting poorer outcomes [18–20]. Still, current use of high b-value DWI does not quantify perfusion and cannot be used to discriminate between the cellular injury of HIE and hypoperfusion related to BD.

DWI measures hydrogen motion in tissues. In addition to intra-/extracellular water self-diffusion, DWI is also affected by bulk water movement (cerebrospinal fluid (CSF)) and intravascular motion [21–23]. While high b-value DWI measures molecular self-diffusion, low b-values are more sensitive to blood flow [21, 23–25]. This is the basis of intravoxel incoherent motion (IVIM), a noncontrast sequence allowing derivation of perfusion (vascular) and diffusion (extravascular) fractions and is quantified by ADC [23] which has been used to evaluate acute stroke, dementia, as well as brain and head and neck masses [26–32]. Though IVIM has many advantages, including its independence from arterial timing and its ability to quantify perfusion without contrast, postprocessing is time consuming and imaging times can be long since multiple b-values are necessary for processing [26, 33]. The current view is that b-value gradients between 0 and 200 s/mm^2^ primarily measure perfusion, whereas gradient strengths greater than 200 s/mm^2^ primarily measure molecular diffusion [22, 34].

We hypothesized that low b-value DWI could also be a helpful diagnostic tool in detecting global hypoperfusion. Specifically, our goal was to determine if low b-value DWI could separate the cellular injury of HIE from the global hypoperfusion of BD. To test this hypothesis, we evaluated patients after cardiorespiratory arrest undergoing MRI with low b-value DWI.

## Methods

This retrospective study was conducted under an Institutional Review Board approved protocol and informed consent was waived. Investigators were compliant with the Health Insurance Portability and Accountability Act and Good Clinical Practice guidelines.

### Subjects

An MRI report search was initiated on all cardiorespiratory arrest patients diagnosed with HIE or BD at our quaternary care center from 2012 to 2018. Inclusion criteria included findings of diffuse hypoxic and/or anoxic injury on MRI and clinical history of arrest. A b50 DWI sequence has been routinely added at our institution as a rapid noncontrast method of perfusion evaluation in patients with stroke within the last 6 years. Only subjects with DWI acquired at both low b-values of 50 s/mm^2^ in three orthogonal planes (TR = 8000 ms, TE = 90 ms, flip angle = 90 degrees, resolution = 2.2 × 2.2 × 11 mm, slice gap = 1 mm, time = 1 min 47 s), and high b-values of 1000–2000 s/mm^2^ in 12 to 20 diffusion direction planes (resolution = 1.8 × 1.8 × 3.3 mm), as well as high-resolution magnetization prepared rapid acquisition gradient echo (MPRAGE) (TR = 1530 ms, TE = 4.04 ms, TI = 950 ms, flip angle =15 degrees, resolution = 1.0 × 1.0 × 1.2 mm) were included in this study. Subjects were excluded for the following: no MRI evidence of hypoxia, primarily embolic disease, the presence of a large vessel infarct, extensive motion or metallic artifact causing image degradation, and lack of or unusable b50 ADC sequence. A formal clinical BD examination was required in all BD subjects. For comparison, MRIs of normal age- and sex-matched subjects were also collected. All cases were scanned on Siemens 1.5 T scanners (Avanto or Aera).

### Clinical review

Each subject underwent a chart review with the following information recorded: age, sex, cause of arrest, time between arrest and return of spontaneous circulation (ROSC), and time between ROSC and MRI. The use of targeted temperature management was recorded for each subject. The blood pressure and EEG findings closest to the time of MRI were recorded. In the case of patient survival, the cerebral performance category was determined.

### Voxel segmentation

To obtain estimates of b50 ADC values localized to brain gray and white matter, MPRAGE segmentation was performed using SPM 12 (Wellcome Trust, London) for MATLAB (Natick, MA). The MPRAGE image was coregistered using rigid transform to the b50 ADC scan for each subject (coregister: estimate) and the MPRAGE image was segmented into gray matter, white matter, and CSF components (segment: 3 tissue classes, thorough clean, native space). The white matter and gray matter images were resampled at the resolution of the coregistered b50 ADC image, and voxels for gray and white matter were included in a restriction mask. ADC values for these voxels were obtained for each BD, HIE, and control subject, and histograms and summary statistics were obtained for these voxels, with scaling of the histogram to peak value (bin size 0.01 from 0 to 3 for b50 ADC value). The ADC values were calculated for gray and white matter at the time of imaging using Siemens online reconstruction algorithm and reported in units of 1000 × ADC, so ADC values obtained from the images were divided by 1000 prior to analysis.

### Statistical analysis

Two-tailed *t* tests were used to determine significance at *p* < 0.05, and logistic regression analyses were performed using Stata 14 (Stata Statistical Software: Release 14; College Station, TX).

## Results

### Subjects

A total of 98 subjects with a clinical history of cardiorespiratory arrest received a brain MRI at our institution from 2012 to 2018. Of these subjects, 53 were excluded from the analysis for the following reasons: 16 demonstrated predominantly embolic disease, 7 had the presence of a large-vessel infarct, 4 had image degradation related to artifact, 8 lacked a b50 ADC sequence, 16 had a normal MRI with no evidence of hypoxic disease, and 2 had MRI and clinical findings consistent with BD but they never underwent a formal BD examination. Forty-five cardiorespiratory arrest subjects met all inclusion criteria for analysis, with all containing b50 ADC imaging and high b-value DWI imaging (36 subjects with b2000, and 9 subjects with b1000 DWI). Of these, 9/45 had a clinical diagnosis of BD and 36/45 HIE (Table 1).

**Table 1:** Demographic and clinical information of the three subject groups

Subject demographics	Brain death	HIE	Normal
*n*	9	36	19
Age, years	27–68 (44 ± 15.9)	22–64 (44 ± 16.5)	21–64 (45 ± 15.1)
Sex, female (F), male (M)	3 F, 6 M	15 F, 21 M	10 F, 9 M
Cardiorespiratory arrest (*n*)	9/9	36/36	0/19
Time to ROSC	3.5–40 min (29.2 ± 13.2)	3–60 min 16.4 ± 15.0)	N/A
Time from ROSC to MRI	0.1–6 days (2.2 ± 1.8)	0.2–16 days (3.8 ± 3.0)	N/A
Blood pressure closest to scan time			
Systolic	81–184 (118.9 ± 33.1)	91–164 (127.5 ± 21.1)	110–144 (127.8 ± 11.5)
Diastolic	52–141 (77.5 ± 28.1)	53–109 (73.6 ± 15.8)	43–91 (75.5 ± 14.1)
Survival (*n*)	0/9	7/36	N/A
b50 ADC – gray matter (in ADC × 10^−3^ mm^2^/s)	0.70 ± 0.18	1.95 ± 0.25	1.79 ± 0.12
b50 ADC – white matter (in ADC × 10^−3^ mm^2^/s)	0.50 ± 0.17	1.44 ± 0.24	1.26 ± 0.14

Values are shown as range (mean ± standard deviation) or mean ± standard deviation unless otherwise indicated

*ADC* apparent diffusion coefficient, *HIE* hypoxic ischemic encephalopathy, *MRI* magnetic resonance imaging, *N/A* not applicable, *ROSC* return of spontaneous circulation

The nine BD subjects included in the study all underwent formal clinical BD examination and, in addition, a board-certified neurologist with experience in BD testing reviewed the patient charts, blinded to neuroimaging findings, to determine the diagnosis of BD using the 2010 American Academy of Neurology criteria [35]. The following findings were documented: examination performed without the presence of CNS depressant medications, pupils fixed and dilated, no corneal reflex, no withdrawal to pain or gag reflex, and no return of neurological function. In two subjects the apnea test was undocumented, and in one subject absence of brainstem reflexes was undocumented on chart review. In two subjects ancillary testing was required and SPECT nuclear scintigraphy was performed to confirm the BD diagnosis.

All HIE subjects had clinical and imaging diagnosis of HIE. Nineteen normal subjects underwent MRI for subjective neurologic symptoms ranging from headache to vertigo. All had a negative neurological and imaging work-up with normal MRI findings for age.

*T* tests demonstrated no significant age difference between BD versus normal (*p* = 0.98) or HIE (*p* = 0.122) groups. Fisher’s exact test also showed no difference in sex proportions between BD versus HIE (*p* = 0.72) or normal (*p* = 0.43) subjects.

There was a poor correlation of time to ROSC and ADC values (*r* = −0.27 in gray matter and −0.29 in white matter) and time between ROSC and MRI with ADC values (*r* = 0.23 in gray matter and 0.27 in white matter). ADC values between gray and white matter regions demonstrated excellent correlation (*r* = 0.98).

### Clinical results

The majority of HIE and BD subjects had a cardiac etiology for arrest (30/45 patients). The remaining subjects had mixed cardiopulmonary or predominantly pulmonary causes for arrest including asthma exacerbation, hanging, status epilepticus, and overdose.

Time from arrest to ROSC was available in 7/9 subjects with BD and 24/36 subjects with HIE. Average time to ROSC was longer in the BD population (29.2 min) compared with the HIE population (16.4 min, *p* = 0.13) (Table 1). Time between ROSC and MRI was shorter in the BD population (2.2 days) compared with HIE (3.8 days, *p* = 0.3). Information on the targeted therapeutic protocol was available in 35/36 HIE patients, with 28 of these patients undergoing this therapy. In the BD population, 7/9 subjects underwent targeted temperature management.

EEG was performed in 32/36 subjects with HIE and demonstrated predominantly global slowing in 23 subjects, with the remainder of subjects having a burst suppression pattern, as well as a myoclonic seizure pattern. Seven BD subjects underwent EEG with all seven demonstrating a flat pattern.

Seven HIE subjects survived after the acute hospital course, with two of these subjects having a Cerebral Performance Category (CPC) score of 2 (good function), and five patients demonstrating a CPC score of 3 or 4 (poor function). There was no significant difference in ADC values between the surviving subjects and the remaining population who died (*p* = 0.19).

### Qualitative MRI findings

**Fig. 1:**
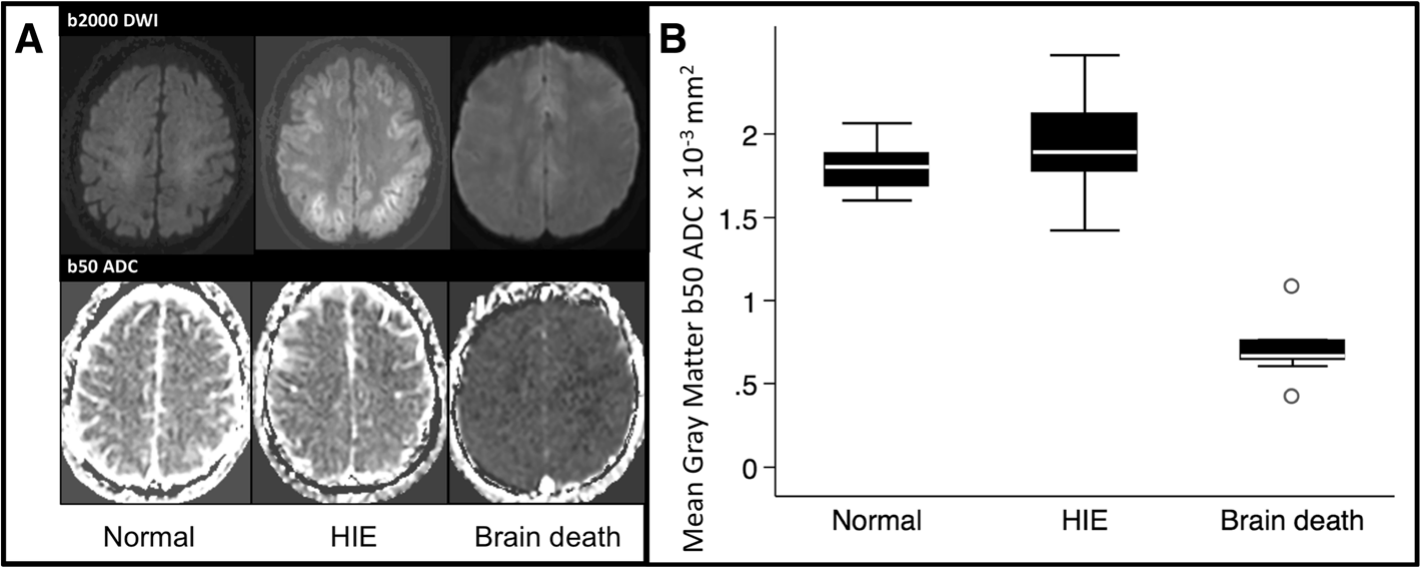
b50 apparent diffusion coefficient (ADC) values in normal, hypoxic ischemic encephalopathy (HIE), and brain death populations. A panel of representative images (**a**) demonstrates high b-value DWI trace images (top) and b50 ADC maps (bottom). In brain death patients, there was diffuse hypointensity on ADC maps compared with normal and HIE subjects. Pooled quantified ADC data (**b**) in box-and-whisker format demonstrate markedly lower mean ADC values in brain death subject gray matter compared with normal and HIE subjects, *p* < 0.001

All BD subjects had diffuse gray matter hypointensity on the b50 ADC maps (Fig. 1a), and 8/9 subjects had diffuse white matter hypointensity. Additional findings supporting BD included global cerebral edema with sulcal effacement, tonsillar herniation, and cellular (high b-value) diffusion restriction consistent with anoxic brain injury, with complete loss of flow voids at the skull base on the T2 weighted spin-echo sequence in 7 out of 9 subjects. In the 2/9 subjects with flow voids, one patient had a very thin residual flow voids in the left parasellar internal carotid artery and bilateral M1 segments, and the second patient had more prominent bilateral parasellar internal carotid artery flow voids with flow extending to the bilateral M3 segments.

All HIE subjects had evidence of molecular diffusion restriction on high b-value (1000 or 2000) DWI. Signal abnormality varied between predominantly cortically based restriction in 11 subjects, predominantly deep gray structure involvement in 5 subjects, and equal involvement of both regions in 21 subjects. Three HIE subjects had early tonsillar herniation without absence of flow voids. Low b-value DWI was not visually different between HIE and normal subjects (Fig. 1a).

### Quantitative differences in ADC values

**Fig. 2:**
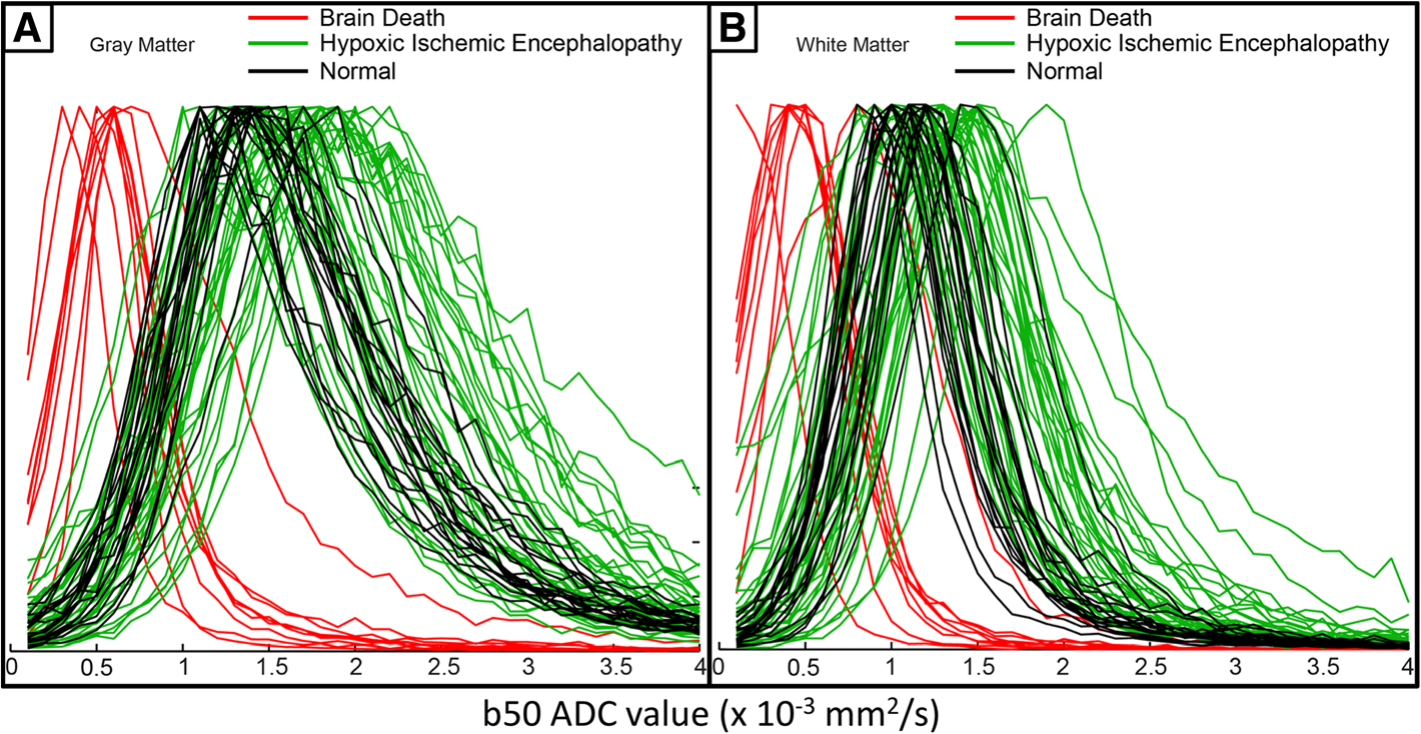
Histogram analysis of groups. Histogram demonstrating the distribution of apparent diffusion coefficient (ADC) values in brain death subjects (red) from normal (black) and HIE subjects (green) in **a** gray and **b** white matter regions

Using automated segmentation to quantify ADC values, BD subjects showed markedly lower mean b50 ADC in gray matter regions compared with HIE (0.70 ± 0.18 vs. 1.95 ± 0.25 × 10^−3^ mm^2^/s, *p* < 0.001) and normal subjects (vs. 1.79 ± 0.12 × 10^−3^ mm^2^/s, *p* < 0.001) (Fig. 1b). In white matter regions, BD subjects also demonstrated markedly lower mean b50 ADC compared with HIE (0.50 ± 0.17 vs. 1.44 ± 0.24, *p* < 0.001) and normal subjects (1.26 ± 0.14, *p* < 0.001). HIE had higher mean b50 ADC compared with normal in gray matter (1.95 ± 0.25 vs. 1.79 ± 0.12 × 10^−3^ mm^2^/s, *p* = 0.016) and white matter (1.44 ± 0.24 vs. 1.26 ± 0.14 × 10^−3^ mm^2^/s, *p* = 0.004). Histogram analysis demonstrated the b50 ADC values in gray and white matter regions in all subjects, with wide separation of values in the gray matter between BD and normal/HIE subjects (Fig. 2). There was predominantly wide separation in white matter between BD and HIE/normal subjects with the exception of one BD patient who demonstrated more elevated white matter values (Fig. 2). Logistic regression analysis of mean ADC value prediction of BD demonstrated perfect prediction with white matter mean ADC < 0.861 × 10^−3^ mm^2^/s, and perfect prediction with gray matter mean ADC < 1.0858 × 10^−3^ mm^2^/s.

## Discussion

Evaluation of patients after cardiorespiratory arrest is a clinical challenge. We found that using a DWI sequence acquired at a single low b-value on a 1.5 T magnet was a promising technique for discrimination of BD from HIE and normal perfusion.

**Fig. 3:**
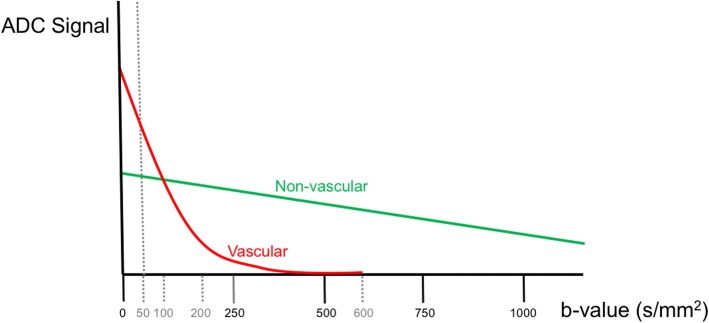
Perfusion and diffusion contributions to apparent diffusion coefficient (ADC) across a spectrum of b-values. Schematic representation of relative contribution of both vascular and nonvascular motion to total ADC decay. The higher ADC vascular compartment is the primary contributor to total decay at gradient strengths less than 100 s/mm^2^, with contribution quickly falling and becoming negligible above a gradient strength of 200 s/mm^2^. Perfusion is the primary contributor to ADC at b50

Results reinforce the concept that low b-values primarily measure brain perfusion properties, rather than diffusion, as has been previously established in the setting of acute stroke (Fig. 3) [26, 29, 30, 32, 36]. Separation of these properties is best seen in the HIE patients who exhibited profound regions of diffusion restriction on b1000–2000 images indicating cellular injury but showed no perfusion restriction on the b50 sequence, in fact showing elevated ADC values in both gray and white matter regions (Fig. 1).

ASL has successfully confirmed BD both quantitatively and qualitatively; however, the necessity for postprocessing and reliance on proper inflow tagging make it less user friendly and potentially less reliable [16, 24, 37]. Because the b50 sequence is “local” in the sense that it provides perfusion information without reliance on these distant processes, it does not suffer from the same potential inaccuracies.

The b50 DWI sequence also has benefits compared with usage of conventional MRI sequences, as it can give quantitative perfusion information compared with the qualitative evaluation of herniation and flow voids. This was reinforced in our results where the b50 ADC values in gray matter corresponded better with clinical diagnosis of BD than the presence of flow voids (which were present at the skull base in 2/9 BD subjects). This single low b-value sequence also has benefits in comparison with IVIM, not least of which are its rapid acquisition time, no need for offline postprocessing, and no specific software as it is a simple modification of a standard three-direction diffusion-weighted sequence. While b50 includes both cellular and vascular information, unlike IVIM which separates these components, it is heavily weighted towards perfusion and therefore able to primarily demonstrate perfusion changes. Visually, this sequence had a distinct appearance in BD with diffuse hypointensity from global perfusion restriction (Fig. 1).

Findings suggest that HIE patients have increased perfusion compared with normal controls, in direct contrast to the global hypoperfusion findings in BD. This is in agreement with prior studies demonstrating hyperperfusion with HIE using both ASL and contrast-based perfusion, reflecting the pathophysiologic course of whole-brain insults where initially there is overcompensation of flow to injured areas [38–43].

**Fig. 4:**
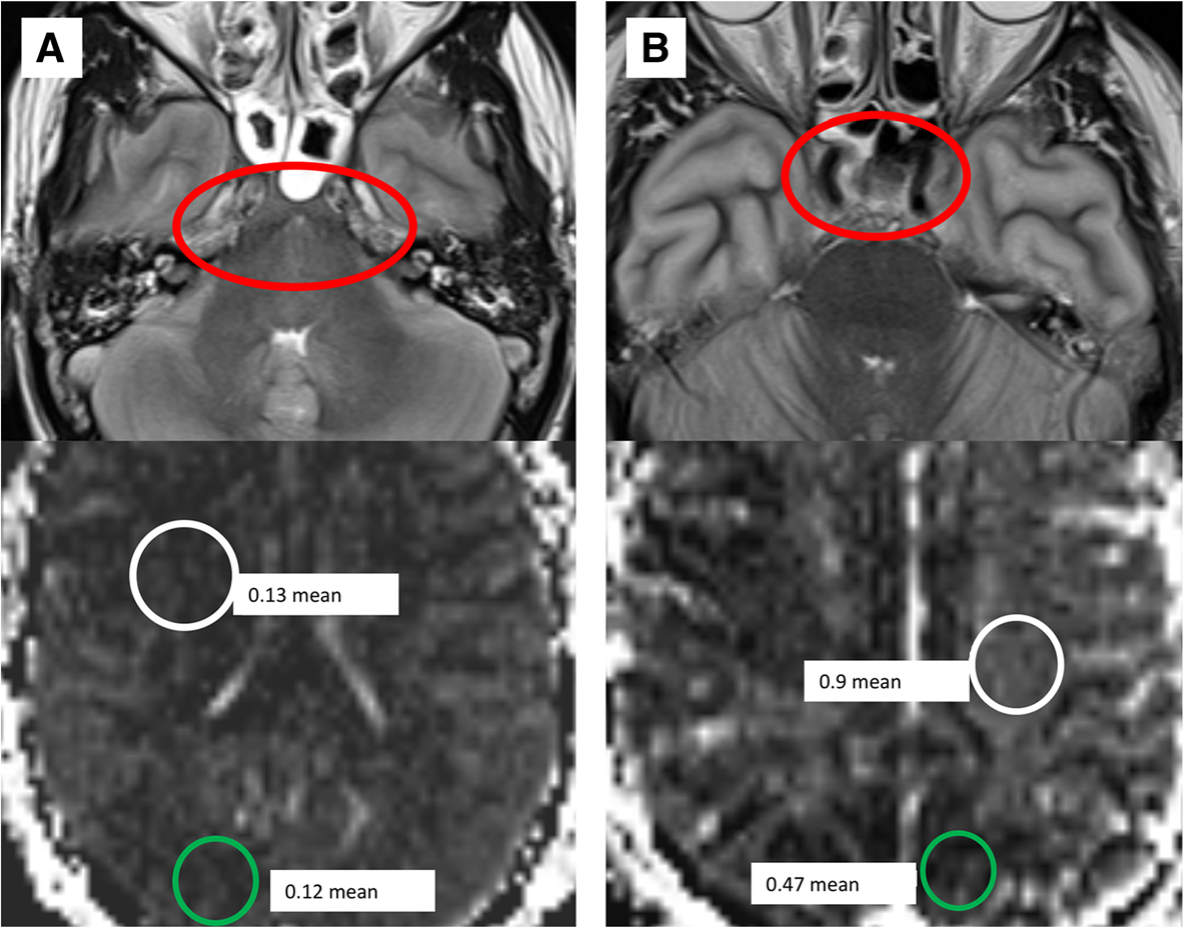
Correlation of hypointensity on b50 ADC maps in the BD population in comparison with flow voids. T2w (upper row) and b50 ADC (lower row) MRI images in two subjects with BD, with **a** subject A demonstrating loss of flow voids, and **b** subject B demonstrating maintained flow voids, at the skull base (red circles). Subject A demonstrated lower white matter (white circle) b50 ADC values than the white matter (white circle) in subject B (0.13 vs 0.92 × 10^−3^ mm^2^/s). The gray matter (green circles) was markedly low in both subjects, although lower in the subject without flow voids (0.12 vs. 0.47 × 10^−3^ mm^2^/s). Both subjects had clinical confirmation of BD per AAN criteria, suggesting that gray matter perfusion values were more predictive of BD than the absence of flow voids

A single outlier was present in the BD population which demonstrated more elevated white matter b50 ADC values (in agreement with HIE/normal values) than the rest of the BD subjects (Fig. 2b). This subject also had mildly elevated gray matter values in comparison with the other BD patients; however, these values were still distinctly lower than those in the HIE/normal subjects (Fig. 2a). This subject demonstrated bilateral parasellar carotid flow voids which extended to the bilateral M3 segments on MRI, although all other MRI findings supported BD (diffuse edema, herniation, and diffusion changes consistent with anoxia). Clinically, this subject had a much shorter timeframe from ROSC to MRI than the other BD subjects, being imaged within 2 h of ROSC as compared with 1–3 days in all other BD patients. The small interval between ROSC and MRI, and the presence of flow voids, support the possibility that this subject had a small residual amount of perfusion at the time of scanning which accounted for the higher b50 ADC values, though no ancillary testing was performed to confirm this. This supports that ADC values in b50 DWI are primarily affected by perfusion, as this patient demonstrated higher values than the BD patients who demonstrated loss of flow voids at the skull base (Fig. 4). This may give insight into the pattern of perfusion loss in BD, with gray matter regions losing perfusion before white matter, although this is only speculative in such a small study population. These findings support that low b50 ADC values in gray matter are more predictive of clinical BD than those in the white matter, and are more predictive of BD than the absence of flow voids at the skull base. A larger study population with confirmatory ancillary testing is necessary to further delineate the optimal b50 ADC threshold for this diagnosis.

Study limitations include vulnerability of echo-planar DWI to motion, chemical shift, susceptibility, and N/2 ghost artifacts, and the use of two different 1.5 T magnets in the study population, all of which could cause inaccuracies in ADC measurements. Additionally, this study is limited by small power and retrospective case-control comparisons. Prospective studies involving larger numbers of BD patients across site, scanner, and sequence architecture are warranted to further establish this technique.

Because BD is primarily a clinical diagnosis, and imaging is only used when the clinical examination cannot be fully obtained or results are in question, the majority of our BD subjects did not have correlative perfusion imaging (7/9). While this is a weakness in terms of validating this particular sequence, low b-value imaging itself has been previously validated as a reliable noncontrast perfusion technique [23–26]. Our study demonstrating wide discrimination of mean ADC values with lack of overlap in the BD group in comparison with the perfused normal and HIE groups in gray matter serves to further support this technique. Although the clinical examination remains the gold standard for diagnosis of BD, this low b-value technique may be a promising supportive tool and deserves further study.

Low b-value DWI is promising for differentiation of BD and HIE with maintained perfusion. This sequence can potentially provide important prognostic information to clinicians and families and aid in critical care decisions. The utility and low-maintenance ability of b50 DWI to demonstrate perfusion abnormalities demonstrate its promise for use in the setting of cardiorespiratory arrest.

## Conclusion

Low b-value DWI is promising for the diagnosis of brain death and HIE after cardiorespiratory arrest. This method demonstrated perfect prediction of brain death using a cutoff ADC value of < 1.085 × 10^−3^ mm^2^/s in the gray matter. Low b50 ADC values in the gray matter were more predictive of brain death than in the white matter.

## Abbreviations

ADC: Apparent diffusion coefficient
ASL: Arterial spin labeling
BD: Brain death
CNS: Central nervous system
CPC: Cerebral Performance Category
CSF: Cerebrospinal fluid
DWI: Diffusion weighted imaging
HIE: Hypoxic ischemic encephalopathy
IVIM: Intravoxel incoherent motion
MPRAGE: Magnetization prepared rapid acquisition gradient echo
MRI: Magnetic resonance imaging
ROSC: Return of spontaneous circulation
SPECT: Single photon emission computed tomography
